# Radiation‐Free In‐Gap Finite Barrier Bound States in a Lithium Niobate‐Based Honeycomb Lattice

**DOI:** 10.1002/nap2.70232

**Published:** 2026-08-05

**Authors:** Yiru Du, Zhixiong Xie, Songyan Hou, Liangliang Liu, Chunyu Huang, Song Zhu, Zhuo Li, Yu Luo, Hao Hu

**Affiliations:** ^1^ National Key Laboratory of Microwave Photonics, College of Electronic and Information Engineering Nanjing University of Aeronautics and Astronautics Nanjing China; ^2^ Photoner Co. Ltd. Cangzhou China; ^3^ Jiangsu Physical Science Research Center National Laboratory of Solid State Microstructures Nanjing China

**Keywords:** finite barrier bound states, photonic crystal waveguides, radiation losses

## Abstract

Finite barrier bound state (FBBS) represents a unique class of boundary modes characterized by negligible inter‐waveguide tunneling, even at subwavelength separations. Although such states hold great promise for suppressing optical crosstalk in integrated photonics, existing FBBS implementations suffer from significant radiation losses because of their eigenfrequencies lying within the bulk continuum. In this work, we introduce a double‐channel waveguide system based on honeycomb photonic lattices made of lithium niobate, which supports FBBSs with eigenfrequencies fully residing within the photonic band gap. This strategic positioning effectively suppresses radiation into bulk modes, enabling low‐loss photon propagation. Moreover, by leveraging the strong electro‐optic response of lithium niobate, we demonstrate dynamic and reversible tuning of the inter‐waveguide tunneling probability via an external voltage. Our platform not only overcomes the key limitations of prior FBBS designs but also offers a robust, tunable architecture for high‐performance on‐chip photonic devices such as lossless modulators, reconfigurable routers, and topological circuits.

## Graphical abstract

This work demonstrates radiation‐free finite barrier bound states (FBBSs), successfully overcoming the high propagation loss challenge inherent in conventional FBBS designs while enabling flexible electro‐optic tunability. These findings establish a versatile platform for on‐chip integrated, high‐modulation‐efficiency electro‐optic modulators and optical switches.

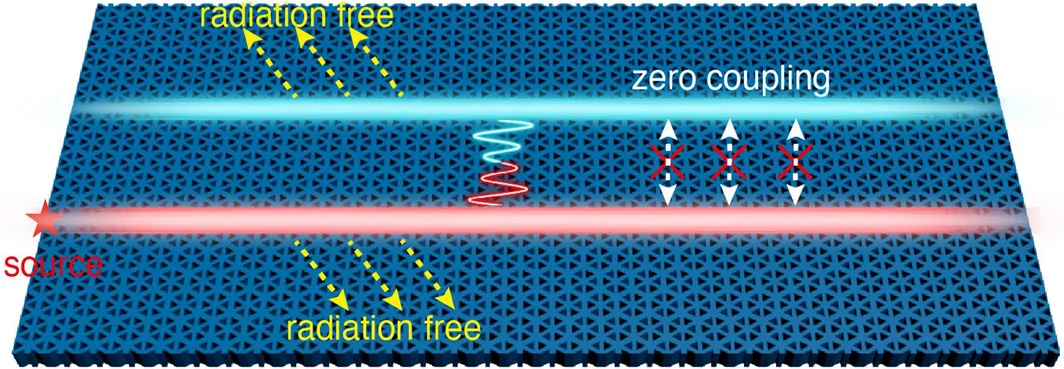

## 1 Introduction

The quantum tunneling effect stands as one of the most profound and counterintuitive manifestations of quantum mechanics, enabling particles to traverse energy barriers that would be impenetrable within the classical physical realm. Since its theoretical inception as an explanation for radioactive alpha decay [1, 2] and its subsequent experimental validation in semiconductor devices [3], this phenomenon has become a cornerstone of modern physics and electronic engineering. This wave‐like ability to traverse barriers finds a direct analogue in photonics, where photons can also tunnel through classically forbidden barriers. When light is confined within subwavelength structures, such as in closely packed waveguides or microcavities in high‐density photonic integrated circuits (PICs), the evanescent fields penetrate physical barriers and lead to uncontrolled energy leakage into adjacent components [4, 5]. Although the tunneling effect underpins the functionality of devices like scanning tunneling microscopes [6], it paradoxically transforms into a detrimental coupling mechanism in the context of integrated photonics, inducing optical crosstalk and imposing a fundamental limitation on further scalability of integration density. For decades, the conventional solution has simply been enlarging the physical separation between components. However, this approach directly contradicts the goals of compact integration.

As photonic technologies advance toward higher integration density and device miniaturization in integrated photonic systems, to realize high‐density integration, various waveguide platforms have been extensively investigated. Recent breakthroughs have demonstrated high‐performance waveguide‐integrated devices, ranging from ultrafast and high‐responsivity photodetectors [7, 8] to extremely compact and multi‐functional components enabled by plasmonic or thermal control mechanisms [9, 10]. Among these, topological photonic crystals (TPCs) [11, 12, 13] have received significant interest for integrated nanophotonics [14, 15, 16, 17, 18, 19, 20, 21, 22]. TPCs formed by periodically repeating unit cells featuring air holes arranged in a chosen lattice symmetry have opened new frontiers for exploring low‐loss waveguides and light–matter interactions [23, 24]. In these systems, waveguide structures are enabled by creating a line defect in the photonic crystal, forming a boundary that serves as a waveguiding path located between the top and bottom photonic crystals [25]. However, even in such topologically robust systems, they still suffer from undesirable inter‐mode coupling because of tunneling effects in miniaturized structures [26, 27, 28, 29]. To suppress the tunneling effect, conventional TPC‐based systems typically require hundreds of lattice periods between adjacent components. In recent years, the rapid development of topological photonics and quantum mechanics provides revolutionary ideas for solving this problem [30, 31, 32]. Among them, Liu et al. proposed the finite barrier bound states (FBBSs) in square photonic crystals, whose tunneling probability vanishes at specific wavevectors even on an extremely small scale [32]. Unfortunately, the eigenfrequencies of the revealed FBBSs fall within the bulk dispersion in their proposals. Fundamentally, radiation‐free FBBS cannot be realized in square lattice systems because of their inherent dispersion characteristics. Such a configuration not only results in the simultaneous excitation of both FBBSs and bulk modes but also introduces high propagation loss because of coupling with bulk states. Moreover, the reliance on perfect electric conductor (PEC) boundaries in previous work [32] severely restricts the application of FBBS in the infrared, terahertz, and optical regimes. This severe propagation loss fundamentally restricts the practical application of FBBS. To address this, TPC structures characterized by complete and tunable photonic band gaps offer an effective solution. However, selecting the appropriate topological phase is critical. Chern insulators are challenging to implement on‐chip as they typically require external magnetic fields to break time‐reversal symmetry [33, 34]. Valley Hall insulators, although passive, rely on breaking spatial inversion symmetry, which inevitably destroys the mirror symmetry prerequisite for sustaining FBBS [35]. In contrast, spin Hall insulators based on the Wu–Hu model are protected by both time‐reversal and spatial inversion symmetries [36, 37]. Crucially, this structure maintains the overall mirror symmetry of the system while offering a tunable photonic band gap, thereby enabling the realization of radiation‐free FBBS.

Another significant limitation of current FBBSs is their static operational frequency in one built system, which constrains their flexibility within reconfigurable photonic circuits. Lithium niobate (LN) is a well‐characterized material whose superior electro‐optic (EO) properties have been extensively leveraged in bulk form for optical communications and radio‐frequency (RF) signal processing [38]. Recent progress in LN nanophotonic devices has unlocked modulation performance in previously unattainable regimes, while simultaneously demonstrating robust potential for large‐scale integration [39, 40, 41, 42, 43, 44]. Furthermore, the substantial performance improvements possible with LN, combined with the relatively mature understanding of the material, could address immediate optical communication demands and enable future sensors and quantum photonic devices [42, 43].

In this work, we reveal the existence of FBBSs in a honeycomb photonic lattice fabricated in LN, and these states efficiently suppress the inter‐waveguide coupling. Different from those in square photonic crystals, our reported FBBSs exhibit an eigenfrequency falling within the photonic band gap. Such a feature favorably avoids the radiation losses of FBBSs into the bulk states induced by fabrication imperfections, enabling a low‐loss transmission. In addition to low transmission loss, our platform exhibits strong tunability of inter‐waveguide coupling by exploiting the high EO coefficient of LN. Our findings are therefore applicable to integrated photonic chips that are both loss‐free and highly efficient.

## 2 Results and Discussion

Without loss of generality, we consider a honeycomb photonic lattice fabricated in a generalized dielectric material (with relative permittivity *ε*_r_) as shown in Figure 1a. Each honeycomb lattice unit cell contains six identical equilateral triangular air holes, where *l* is the side length of each triangle, *a* is the lattice constant, and the distance *D* between the center of each triangle and the honeycomb lattice center is uniform. The defect waveguide constructed upon this honeycomb photonic lattice platform exhibits robust stability, remaining highly resilient to diverse perturbations including variations in the dimensions and positions of the triangular air holes. We use finite element analysis (COMSOL Multiphysics) to calculate the band structures of the honeycomb photonic lattice. In this work, we focus on transverse‐electric (TE) modes, where the electric field vector lies in the *x*–*y* plane, whereas the magnetic field vector is oriented along the *z*‐axis. As plotted in Figure 1b,c, the Dirac cones from the K and K′ points of the first Brillouin zone fold at the *Γ*‐point when *D* = *a*/3, forming a double Dirac cone (see Figure 1b). Notably, modifying *D* would open a photonic band gap near the Dirac frequency at the *Γ*‐point without breaking the *C*_6_ rotational symmetry of the honeycomb photonic lattice (see Figure 1c for *D* = *a*/3.19). To create a waveguide channel, we introduce defects arranged in the *x*‐direction, that is, the elimination of an entire row of triangular holes along the *x*‐direction. The width of the waveguide is defined as *m*. Without a particular statement, the parameters here and below are *ε*_r_ = 4.84, *m* = 132 nm, *a* = 445 nm and *l* = 140 nm. The schematic diagram in Figure 1a illustrates the fundamental mechanism of the FBBS. Protected by the mirror symmetry of the structure, the wavefunctions of the boundary modes penetrating into the bulk region exhibit an oscillating decaying nature characterized by a sine‐like function. At specific wavevectors, this unique oscillating feature leads to a strictly zero coupling strength between the top and bottom waveguide channels. At these nodal points, the even and odd modes become degenerate, and the evanescent fields from the two boundaries are perfectly decoupled, which gives rise to the FBBS.

**FIGURE 1:**
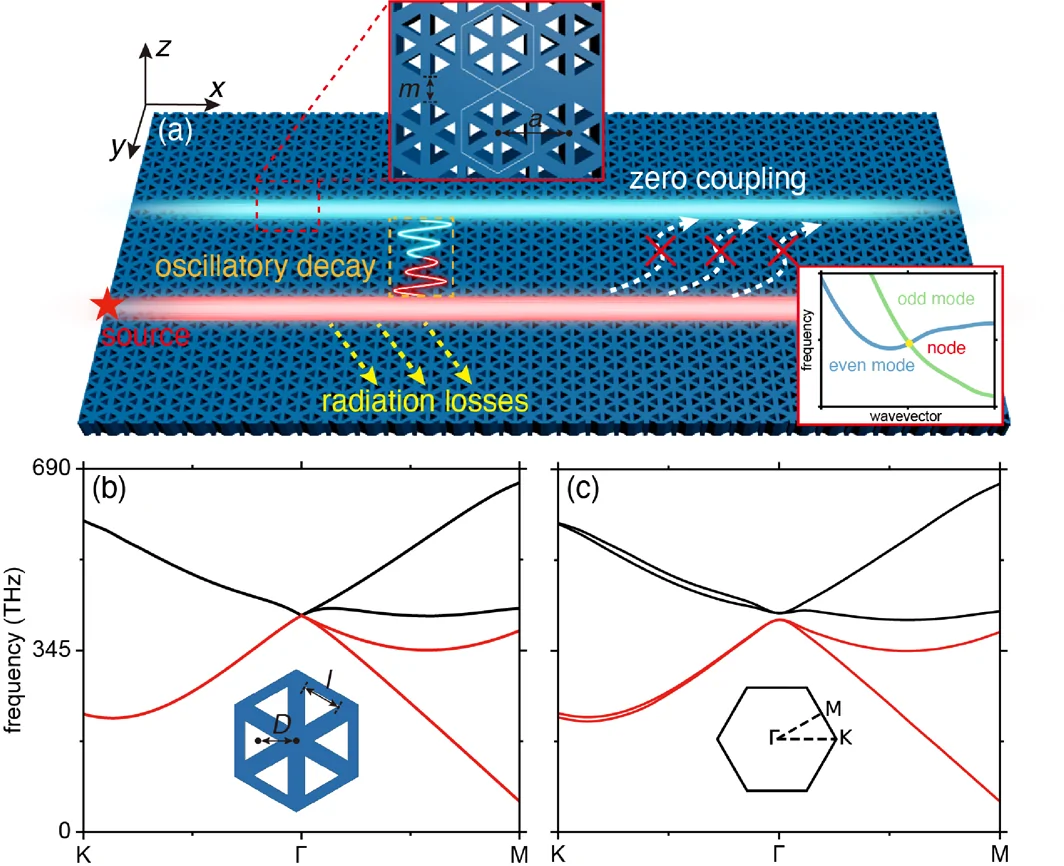
Design of waveguide structure based on honeycomb photonic lattices. (a) Schematic diagram of the photon system and FBBS. A waveguide structure is formed by removing an entire row of triangular holes from the honeycomb photonic lattice system. The white line represents the honeycomb lattice unit cell. Here, *m* is the waveguide width, *l* is the side length of the triangle, *a* is the lattice constant, and *D* is the distance between each triangle and the unit cell center. The adopted parameters are as follows: *m* = 132 nm, *a* = 445 nm, and *l* = 140 nm. Inset: the illustrate of degenerate between odd and even mode, which induced the FBBS. (b, c) Honeycomb lattice's band diagrams for different distance *D*. In (b), *D* = *a*/3. Inset: the structure dimensions of the honeycomb lattice unit cell. In (c), *D* = *a*/3.19. Inset: Brillouin zone of the honeycomb lattice.

Second, we demonstrate that edge states appear in the waveguide structure. To visualize the edge states, we plot the dispersion of edge states in Figure 2a. We can see that the single‐channel waveguide structure supports three edge states, that is, Edge State 1, Edge State 2, and Edge State 3, whose field distributions are shown in the inset of Figure 2a, respectively. We would like to emphasize that when one additional waveguide is introduced into the system, the eigenmodes in this double‐channel waveguide system will interact and split into even and odd modes with different frequencies. This splitting frequency is the origin of energy oscillation between waveguides; the coupling strength (and thus the energy oscillation rate) between waveguides is directly proportional to the magnitude of this splitting. We consider Edge State 1 as an example (see Figure 2b), where the separation between two neighboring waveguides is 2 μm. The dispersions of odd and even modes intertwine with each other, inducing four dispersion nodes in the band diagram. At these node frequencies, the frequency splitting vanishes, resulting in the degeneracy of the even and odd modes. Consequently, the coupling strength drops to zero, implying an infinite energy oscillation period. This means the modes at these four nodes are dynamically prohibited from tunneling to the adjacent waveguide, known as FBBSs [32]. The node frequency corresponds to the eigenfrequency of FBBS. We emphasize that the number of nodes equals the lattice periods between adjacent waveguides. Furthermore, Figure 2b highlights a distinct advantage of our defect‐based photonic crystal waveguide system compared to previous works, in which the eigenfrequency of FBBS is located in the continuum. As shown, the eigenfrequency of our FBBS resides in the discrete domain below the light cone. This spectral positioning ensures that the FBBS in our system lacks coupling channels for radiation into the air, thereby enabling lossless transmission without the need for parallel waveguides. This radiation‐free capability is a feature that topological domain wall structures cannot achieve.

**FIGURE 2:**
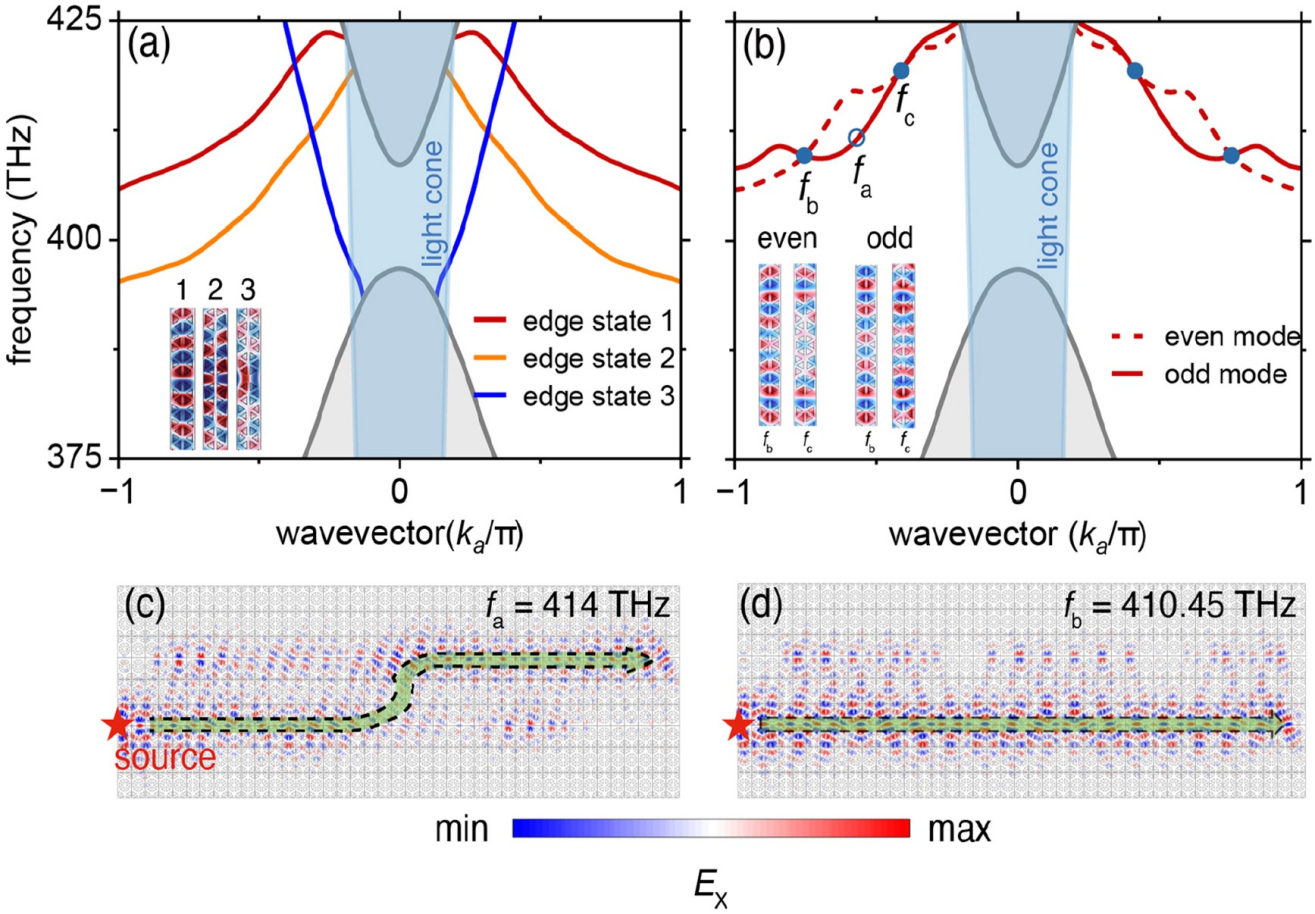
Comparison of FBBSs and traditional edge states in coupled waveguide systems. (a) Band diagram of the single‐channel waveguide structure. The red, orange, and blue lines represent the dispersions of Edge States 1, 2, and 3, respectively, whose electric field distributions are shown in the inset. (b) Band diagram of the double‐channel waveguide structure. The solid and dashed lines represent the odd and even modes of Edge State 1, respectively, whose electric field distributions are shown in the inset. The nodes of dispersion are marked by solid blue points. In (a, b), the blue area corresponds to the light cone and the grey area corresponds to bulk bands. (c, d) Electric field distributions excited by a dipole source (marked by red stars) in a double‐channel waveguide structure. In (c), the adopted frequency is *f*_a_ = 414 THz; in (d), the adopted frequency is *f*_b_ = 410.45 THz.

To further illustrate the vanished coupling strength of FBBS, we calculate the field distributions of edge states in the double‐channel waveguide structure in Figure 2c,d. The edge state is excited by a dipole source (marked by the red star) located at the input port of the bottom waveguide channel. In Figure 2c, the excitation frequency *f*_a_ = 414 THz is off the node frequency; in Figure 2d, the excitation frequency *f*_b_ = 410.45 THz is exactly the node frequency. The results show that the edge states excited at a non‐node frequency (i.e., *f*_a_) propagate along the bottom waveguide and gradually couple to the top one as the edge states propagate forward (Figure 2c). In sharp contrast, the excited edge states are well confined at the bottom waveguide at *f*_b_, reflecting the decoupling nature of FBBSs (Figure 2d). The residual leakage electric field observed in the upper waveguide in Figure 2d, even at the FBBS frequency, is attributed to the simultaneous excitation of Edge States 2 and 3 alongside Edge State 1. Because the odd and even modes of these latter states are not degenerate at this frequency, they induce energy coupling into the upper waveguide.

Next, we reveal that significant suppression of FBBS radiation loss in the system can be achieved through reasonable geometric design (e.g., waveguide width). This is because the node frequency of FBBSs initially overlapping with the bulk dispersion can be shifted into the band gap by varying the waveguide widths (Figure 3d). When Δ*m =* 0, the node frequency (as marked by character A in Figure 3a) falls within the bulk dispersion. In this case, the FBBS is likely to couple to bulk states in the presence of fabrication imperfections, inevitably leading to radiation losses and reducing transmission efficiency (Figure 3e). If Δ*m* increases to 0.02*a*, the node frequency decreases to the bottom of the upper‐branch bulk dispersion. If Δ*m* further increases to 0.05*a*, the node frequency is completely separated from the bulk dispersion. In this circumstance, the FBBS has no chance to be coupled into bulk states, thereby exhibiting negligible radiation losses (Figure 3f). To quantitatively compare the radiation losses of in‐gap and out‐of‐gap FBBSs, we plot in Figure 3g by extracting the electric field intensity along the bottom waveguide in Figure 3e,f. Clearly, as the propagation distance increases to 30 μm, the field intensity of the in‐gap FBBS decreases by only 30%, whereas that of the out‐of‐gap FBBS drops by more than 60%. In practical nanofabrication, geometric imperfections are typically inevitable, which may affect the behaviors of FBBS. However, such influence could be effectively suppressed by introducing suitable fabrication techniques, such as focused ion beam (see detailed discussions in Supporting Information S1: Section S1).

**FIGURE 3:**
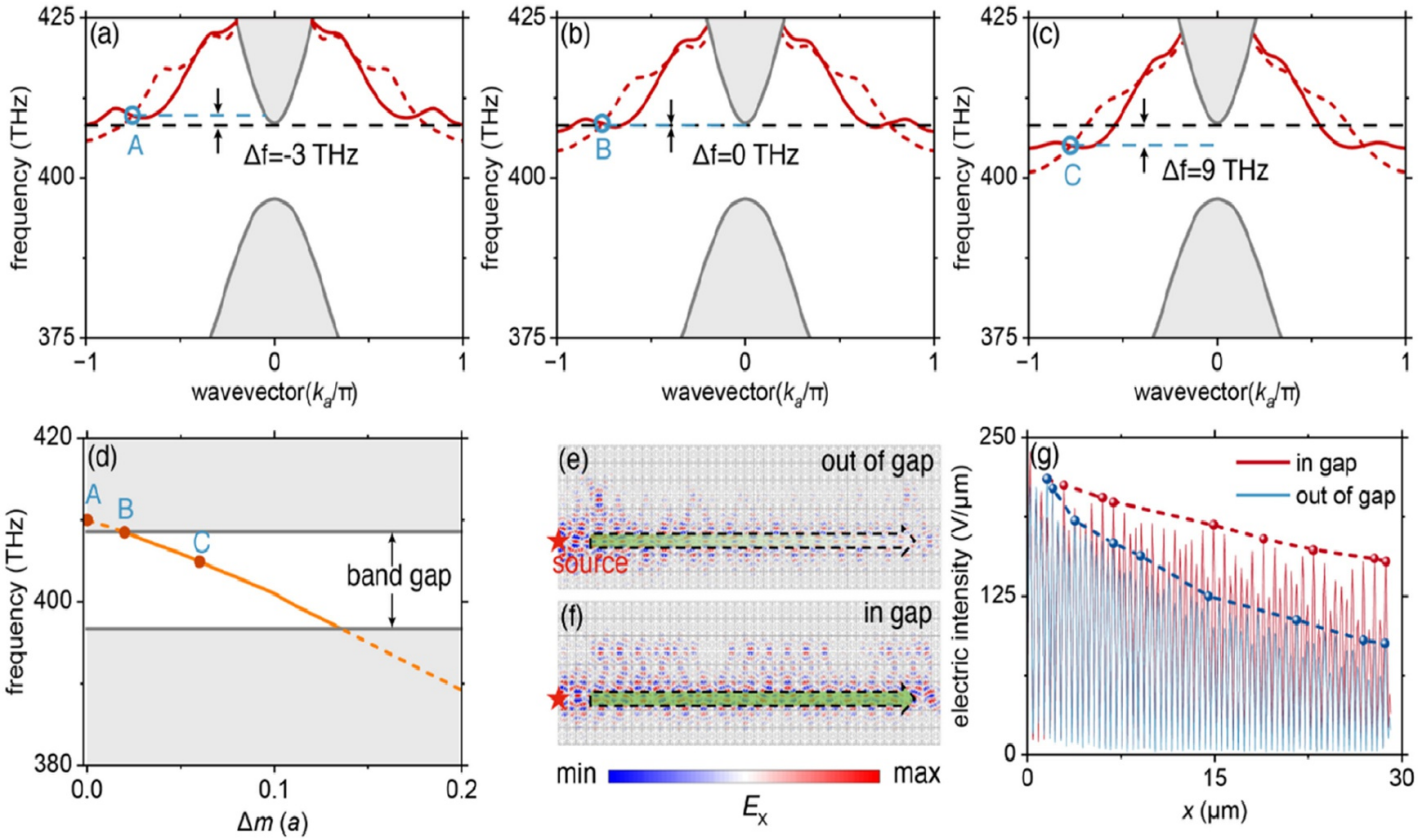
Influence of waveguide width on the behaviors of FBBSs. (a, b, c) Band diagrams of a double‐channel waveguide structure with different waveguide widths. In (a), Δ*m* = 0; in (b), Δ*m* = 0.02*a*; in (c), Δ*m* = 0.05*a*. The red line corresponds to the odd and even modes of Edge State 1; the blue circle corresponds to the eigenfrequency of FBBS; the grey area corresponds to bulk bands. (d) Node frequency as a function of Δ*m*. Points A, B, and C correspond to the band diagrams shown in (a), (b), and (c), respectively. (e, f) Comparison of the electric field intensities of the edge state in the waveguide under different Δ*m* and frequencies. In (e), *f*_1_ = 410 THz and Δ*m* = 0; In (f), *f*_2_ = 399 THz and Δ*m* = 0.05*a*. (g) Electric field intensity at the center of the bottom waveguide as a function of propagation distance. The red curve represents the state with node frequency that lies within the photonic band gap, and the blue curve corresponds to the state with a node frequency outside the photonic band gap.

Our double‐channel waveguide structure not only exhibits minor radiation losses, but also features controllable inter‐waveguide coupling by adopting nonlinear materials. To demonstrate this, we replace the host material with *x*‐cut LN, where the *n*_y_ = *n*_e_ is the extraordinary refractive index and *n*_x_ = *n*_z_ = *n*_o_ is the ordinary refractive index. LN has a high EO efficiency, whose extraordinary refractive index *n*_e_ changes with the relation:

(1) $$\Delta n=-\frac{1}{2}n_{e}^{3}\gamma_{33}E_{y}$$

*E*_y_ = *U*_y_ */d* is the *y*‐direction electrostatic field induced by applying an external voltage *U*_y_ across a pair of electrodes separated by *d*. Moreover, *γ*_33_ is the EO coefficient for extraordinary polarization. The adopted parameters are as follows: *n*_e_ = 2.15, *γ*_33_ = 30.4 pm/V and *d* = 6.2 μm. A capacitively loaded traveling‐wave electrode design is adopted to overcome potential electrical parasitic effects (see Supporting Information S1: Section S3). The relation between the refractive index variation and the external voltage is demonstrated by the red line in Figure 4a. The refractive index variation impacts the eigenfrequency of FBBS, thereby affecting inter‐waveguide coupling at a given frequency. For instance, inter‐waveguide coupling is zero if *U*_0_ = 0 V (i.e., without external voltage) and *f* = 399 THz (i.e., the eigenfrequency of FBBS). This can be learned from Figure 4b, where edge states excited by a dipole source are solely localized at the bottom waveguide [also see the negligible electric field (the blue line in Figure 4a) at the position marked by the blue circle in Figure 4b in the top waveguide]. However, the inter‐waveguide coupling is significant at the same frequency when the voltage is *U*_1_ = 9.3 V, because the applied voltage increases the eigenfrequency of FBBS. In this case, the edge states in the bottom waveguide rapidly transfer into the top waveguide, with the electric field in the top waveguide significantly enhanced. Further increasing the external voltage makes the inter‐waveguide coupling even stronger (see blue line in Figure 4a). To further demonstrate the tunability advantages of the proposed structure, we plot the normalized transmission spectra of the upper waveguide channel under various applied voltages (Figure 4d). Without an external voltage, the transmission exhibits a pronounced dip around 410.5 THz (i.e., the eigenfrequency of the FBBS), which verifies the complete suppression of inter‐waveguide coupling. In contrast, upon applying an external voltage, the frequency corresponding to the transmission minimum shifts in tandem with the eigenfrequency of the FBBS, thereby enabling dynamic manipulation of the inter‐waveguide coupling across a terahertz‐scale bandwidth. However, utilizing a photonic crystal architecture inevitably increases the device thickness compared to mainstream thin‐film lithium niobate modulators, which may impose constraints on the ultimate modulation speed. The extreme sensitivity of the coupling strength to frequency shift uniquely facilitates extremely high modulation efficiency. As depicted in Figure 4e, the eigenfrequency shift of the FBBS exhibits a perfectly linear dependence on the applied voltage, owing exclusively to the Pockels electro‐optic effect of lithium niobate. The modulation efficiency, defined by the slope of this linear fit, reaches an impressive value of 3.37 GHz/V. However, the extreme sensitivity also makes the device susceptible to ambient temperature fluctuations. Detailed analysis regarding the influence of environmental thermal noise on the modulator is provided in Supporting Information S1: Section S2.

**FIGURE 4:**
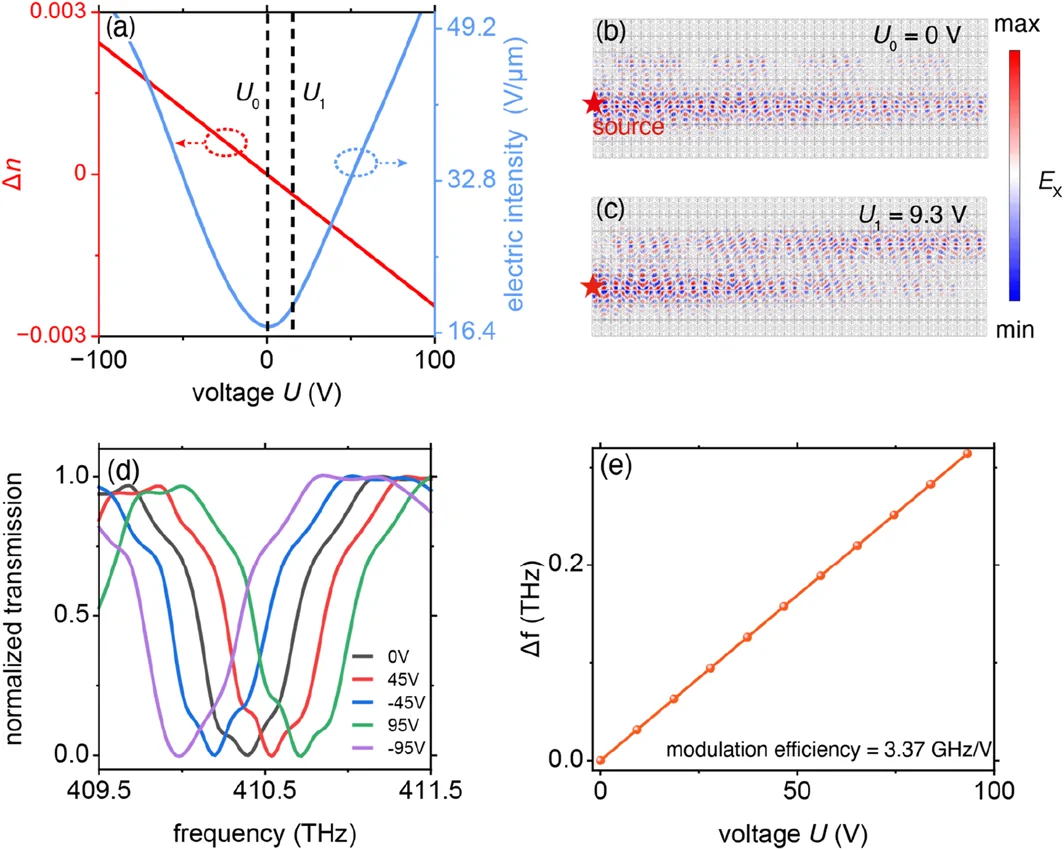
Engineering coupling strength in double‐channel waveguide structure made of LN with external voltages. (a) Refractive index variation and electric field intensity at the position denoted by the green circle in (b, c) as a function of applied voltage. (b, c) Electric field distribution excited by a dipole source in the double‐channel waveguide structure. In (b) *U*_0_ = 0 V; in (c) *U*_1_ = 9.3 V. The studied Δ*m* is 0.05*a*, and the adopted frequency in (b, c) is 399 THz. (d) Normalized transmission spectra of the upper channel in the double‐channel waveguide structure excited by a point dipole source under various applied voltages. (e) Eigenfrequency shift of the FBBS as a function of the external voltage. The modulation efficiency corresponds to the slope of the plotted line.

## 3 Conclusion

In conclusion, we propose a novel LN waveguide structure that enables loss‐free and tunable photonic transport based on photonic crystals. Numerical simulations confirm the existence of FBBSs in a double‐channel waveguide system, which efficiently suppresses the coupling between edge states at an ultra‐compact scale. By judiciously adjusting the waveguide geometry, we successfully tune the eigenfrequency of FBBS into the photonic band gap, thus enabling long‐distance propagation of edge states. This strategy offers an effective and versatile solution for simultaneously suppressing detrimental crosstalk and radiation losses in micro/nano devices. Moreover, by leveraging the Pockels effect in LN, we demonstrate full controllability of inter‐waveguide coupling, allowing for efficient near‐field photonic routing. Furthermore, extending this approach to higher‐density multi‐channel arrays presents exciting prospects. Supporting Information S1: Section S4 provides a detailed discussion of the channel‐selective coupling characteristics of the FBBS in multi‐channel systems, which may find applications in reconfigurable optical routing and programmable photonic circuit networks.

## Author Contributions

**Yiru Du:** software, validation, visualization, writing – original draft, writing – review and editing, methodology, conceptualization, data curation, investigation. **Zhixiong Xie:** writing – review and editing, visualization, formal analysis, supervision, validation, investigation. **Songyan Hou:** supervision, writing – review and editing, methodology, conceptualization, investigation. **Liangliang Liu:** writing – review and editing, supervision, project administration, visualization. **Chunyu Huang:** writing – review and editing, project administration, visualization, supervision. **Song Zhu:** supervision, project administration, visualization, writing – review and editing. **Zhuo Li:** writing – review and editing, visualization, project administration, supervision. **Yu Luo:** resources, supervision, writing – review and editing, visualization, funding acquisition, methodology, validation, conceptualization. **Hao Hu:** resources, supervision, writing – review and editing, visualization, methodology, funding acquisition, conceptualization.

## Funding

This work is partially sponsored by National Natural Science Foundation of China (Grant No. 12404363); Natural Science Foundation of Jiangsu Province (Grant Nos. BK20241374, SBK20250407355, BK20233001); Distinguished Professor Fund of Jiangsu Province.

## Conflicts of Interest

The authors declare no conflicts of interest.

## Supporting information


Supporting Information S1

## Data Availability

The data that support the findings of this study are available from the corresponding author upon reasonable request.
