# Supplementary material for: Radiation‐Free In‐Gap Finite Barrier Bound States in a Lithium Niobate‐Based Honeycomb Lattice
Source: Nanophotonics. 2026 Aug 5;15(15):e70232. doi: 10.1002/nap2.70232 (PMC13642477; doi:10.1002/nap2.70232)
Supplement: Supplementary file 1 [file NAP2-15-e70232-s001.docx]

Supplementary Information for

“**Radiation-Free In-Gap Finite Barrier Bound States in a Lithium Niobate-Based Honeycomb Lattice**”

Yiru Du, Zhixiong Xie, Songyan Hou, Liangliang Liu, Chunyu Huang, Song Zhu, Zhuo Li, Yu Luo, and Hao Hu

**Supplementary Information Guide:**

-- Section S1. Preparation and Fabricate of LN Photonic Crystals

--Section S2. Influence of Environmental Factors on FBBS

--Section S3. Electrode Design and Mitigation of Electrical Parasitics

--Section S4. Physical Characteristics of FBBS in Multi-Channel Systems

-- Supplementary References

**Section S1. Preparation and Fabricate of LN Photonic Crystals**

The LN thin films required for this work can be prepared by slicing commercial X-cut LN wafers using the Smart-Cut technique. This technology is relatively straightforward and capable of producing high-quality LN thin films with excellent thickness uniformity.

Following the preparation of the LN thin film, nanofabrication is required to etch the triangular air holes. This defines the photonic crystal structure to control the photonic bandgap and forms line-defect waveguides to support the propagation of edge states. Common LNOI etching techniques, such as reactive ion etching (RIE) or inductively coupled plasma reactive ion etching (ICP-RIE), are widely applied. However, the pure physical bombardment nature of these methods, along with the low volatility of the redeposition, inevitably leads to mask edge degradation and byproduct redeposition on the sidewalls. Consequently, traditional ICP-RIE typically results in a significant sidewall inclination angle (deviating from the vertical direction by roughly 15° to 25°) and a high surface roughness on the order of tens of nanometers [1]. This level of sidewall roughness introduces severe optical scattering loss at the boundaries of the triangular air holes, significantly reducing the quality factor of the modes and increasing the propagation loss of the defect waveguide. Meanwhile, a large sidewall inclination angle causes the overall effective refractive index of the photonic crystal to deviate from theoretical values, leading to eigenfrequency shifts and mode splitting, which severely compromise the optical performance. Furthermore, since the FBBS is protected by the structural mirror symmetry, excessive geometric defects may break this symmetry and perturb the underlying physics of the FBBS. Nevertheless, micro-nano optical processing implemented by focused ion beam (FIB) technology can effectively resolve these fabrication-induced issues. Owing to its maskless direct-writing capability and high-precision physical sputtering, FIB can circumvent the process uncertainties inherent in chemical etching. This technique can control the sidewall inclination angle to within 5° [2] and limit the surface roughness to below 3 nm, or even down to the sub-nanometer scale [3]. For the approximately 700 nm wavelength range focused on in this work, the fabrication errors introduced by the FIB process are entirely negligible. Therefore, adopting FIB as the practical nanofabrication method can effectively eliminate the performance degradation caused by sidewall inclination and surface roughness.

On the one hand, perfect triangular geometries are physically unattainable in actual fabrication; In other words, corner rounding (fillets) inevitably exists. To evaluate the impact of this geometric imperfection on the FBBS and its propagation properties, we introduced fillets into the triangular air holes and investigated their effects on the eigenfrequency and propagation loss, as shown in Fig. S1. Here, *r* represents the fillet radius，and the *r* denotes the side length of the triangular air hole. Fig. S1(a) depicts the dependence of the FBBS eigenfrequency on the ratio *r*/*l*。The introduction of fillets alters the effective refractive index of the photonic crystal, resulting in a red-shift of the mode's eigenfrequency. A maximum eigenfrequency shift of 6.2 THz is observed when *r*/*l* = 0.1. To further investigate the impact of corner rounding on the actual propagation characteristics and the coupling between waveguides, we calculated the evolution of the optical intensity at the waveguide center with respect to the propagation distance for different fillet radius (Fig. S1(b)). Over a propagation distance of 17 μm, the condition *r*/*l* = 0.1，induces a maximum propagation loss of approximately 20%. However, the electric field evolutions for *r*/*l* = 0.1 and *r*/*l* = 0.05 are highly consistent with the ideal case without fillets. This demonstrates that the introduction of fillets only induces a specific amount of propagation loss without disrupting the fundamental physics of the FBBS. It can be concluded that a fillet radius within 0.1 times the side length of the triangle will not destroy the core characteristic of the FBBS, which guarantees complete decoupling over sub-wavelength distances. By precisely controlling the gallium ion dose, the FIB technique can easily restrict the fillet radius to around 10 nm, satisfying the geometrical precision requirements of most structural dimensions.

**Fig. S1 Influence of the fillet radius on the FBBS**. (a) The impact of the fillet radius on the FBBS eigenfrequency. Inset: Schematic of the triangular air hole with corner fillets.  (b) Electric field intensity at the center of the waveguide as a function of propagation distance with different fillet radius. The orange curve represents the *r*/*l* = 0, the blue curve corresponds to the *r*/*l* = 0.05, and the red curve represents the *r*/*l* = 0.1. The excited frequency for different fillet radius is 410.5 THz, 410 THZ and 404.3 THz respectively. Here, *r* is the fillet radius and *l* is the side length of triangle.

**Section S2. Influence of Environmental Factors on FBBS**

In a cleanroom environment, temperature fluctuation is the dominant factor affecting the refractive index of LN. The sensitivity of the FBBS to material refractive index changes implies that minute temperature variations could impact the modulation efficiency and stability of the FBBS-based LN modulator. Since the ordinary refractive index changes with temperature are almost negligible, here we only consider the effects on the extraordinary refractive index. The temperature dependence of the extraordinary refractive index(*n*_e_) is governed by this equation [4],[5]:

$n_{e}^{2}\left( \lambda,T \right)=A+\frac{B+b\left( T \right)}{\lambda^{2}-\left( C+c\left( T \right) \right)^{2}}+\frac{D}{\lambda^{2}-E}-F\lambda^{2}$(S1)

where *a*(λ) represents the thermophotonic effect term and *b*(λ) is the temperature correction term; *S*(λ) is the dispersion term and *λ* is the wavelength. Since the FBBS in our study fundamentally operates at a specific frequency point, taking the derivative of Eq. (S1) with respect to temperature at a fixed wavelength yields the extraordinary thermo-optic coefficient $\frac{dn_{e}}{dT}$，Consequently, the temperature-induced variation in the extraordinary refractive index can be simplified as:

$\Delta n_{e}=\frac{dn_{e}}{dT}\cdot\Delta T$	(S2)

where *ΔT* is the temperature variation. In the 700 nm wavelength range, the extraordinary thermo-optic coefficient of LN is approximately 1.2×10^−4^ K^-1^. The ambient temperature in a cleanroom is typically maintained at 22℃±1℃, which yields a maximum index change of $\Delta n_{e}\approx$ 2.4×10^-4^. This value is comparable to the index change induced by an applied voltage of 9.3 V in our design, which could severely compromise the operational stability of the LN modulator. To counteract the refractive index variations induced by ambient temperature fluctuations in experiments, a practical and widely adopted solution is the integration of a thermoelectric cooler (TEC). By forming a closed-loop PID control system with a thermistor, the TEC can precisely anchor the chip temperature within ±0.1 °C, corresponding to an index variation of only Δ*n*_e_ ≈ 2.4×10^-5^, which causes a global shift in the eigenmode frequency of roughly 2 GHz, generating a comparable even-odd mode frequency splitting. Based on this splitting, the theoretical coupling length is calculated to be only about 1 cm through coupled-mode theory. For a micro-scale LN modulator, the impact of such a long coupling length is entirely negligible.

**Section S3. Electrode Design and Mitigation of Electrical Parasitic**

The high-efficiency LN modulator proposed in this study based on FBBS requires the distance between the external electrodes to be precisely controlled at 6.2 μm. At such a microscopic electrode gap, traditional integrated continuous electrodes inevitably induce severe electrical parasitics. Specifically, the parasitic capacitance significantly slows down the microwave phase velocity, leading to a severe mismatch between the microwave phase velocity and the optical group velocity, which severely limits the operational bandwidth of the device. Additionally, parasitic resistance (aggravated by the skin effect) increases the microwave transmission loss.

To overcome these physical bottlenecks, a capacitively loaded traveling-wave (CTW) electrode (also known as segmented electrodes) is employed. The CTW configuration can be integrated alongside the photonic crystal waveguide without significantly perturbing the optical mode. This advanced electrode arrangement can perfectly mitigate RC limitations to eliminate the adverse effects of electrical parasitics on the modulator's bandwidth and efficiency. It excellently satisfies the high-speed modulation requirements of 3.37 GHz/V proposed in this study [6]. The specific layout of the CTW electrode is illustrated in Fig. S2.

**Fig. S2 Traveling-wave integrated LN modulator with segmented electrodes.** (a) 3D artistic schematic of the overall modulator design. (b) Cross-sectional schematic of the modulator with segmented electrodes.

**Section S4. Physical Characteristics of FBBS in Multi-Channel Systems**

While the physical characteristics of FBBS in a dual-channel system were discussed in detail in the main text, here we extend the discussion to multi-channel systems and calculate the transmission electric field distributions. We must emphasize that the FBBS is a physical phenomenon specifically tailored for double channel systems. In multi-channel systems, the existence of higher order couplings, such as next nearest neighbor (NNN) coupling, precludes the existence of an ideal FBBS.

Taking an equally spaced triple-channel system as an example, when an excitation source with *f*_0_ = 410.5 THz (i.e., the FBBS frequency in the double channel system) is applied to the bottom waveguide, the coupling to the middle waveguide is strongly suppressed because the nearest neighbor coupling strength is zero. However, energy still transfers to the top waveguide due to the inherent influence of the NNN coupling, as shown in Fig. S3.

**Fig. S3** Electric field distribution excited by a dipole source with an adopted frequency of *f*_0_ = 410.5 THz in an equally spaced triple-channel system.

Conversely, for the middle waveguide, no NNN coupling channels exist. When the same excitation source (*f*_0_ = 410.5 THz) is applied, the energy remains completely confined within the middle waveguide and cannot couple to either the top or bottom side (Fig. S4(a)). When the excitation frequency is shifted to *f*_1_ = 414 THz, the energy couples uniformly to both side waveguides (Fig. S4(b)). Furthermore, the multi-channel system introduces a new degree of freedom: the waveguide spacing. Modifying the spacing between waveguides directly alters the eigenfrequency of the FBBS between those specific two channels. When the distance between the middle waveguide and the two side waveguides differs, the distinct FBBS eigenfrequencies can be exploited to achieve optical beam splitting. To further illustrate this beam-splitting functionality, we calculated the transmission electric fields in an unevenly spaced triple-channel system (Figs. S4(c) and S4(d)). In this setup, the spacing between the middle and top waveguides is 2 lattice periods, whereas the spacing between the middle and bottom waveguides is 4 lattice periods. In Fig. S4c, the excitation frequency *f*_2_ = 415 THz is the eigenfrequency of FBBS between middle and bottom waveguides. In Fig. S4d, the excitation frequency *f*_0_ = 410 THz is the eigenfrequency of FBBS between middle and top waveguides. The results clearly demonstrate that at the excitation frequency of *f*_2_, the edge states decouple from the bottom waveguide and couple exclusively to the top waveguide. Conversely, at *f*_0_, the behavior is exactly the opposite. This physical mechanism effectively realizes a highly selective beam-splitting function.

**Fig. S4 Physical characteristics of FBBS in triple-channel systems.** (a, b) Electric field distributions excited by a dipole source in equally spaced triple-channel system. In (a), the adopted frequency is *f*_0_ = 410.5 THz; in (b), the adopted frequency is *f*_1_ = 414 THz. (c, d) Electric field distributions excited by a dipole source in uneven spacing triple-channel system. In (c), the adopted frequency is *f*_2_ = 415 THz; in (d), the adopted frequency is *f*_0_ = 410.5 THz.

**Supplementary References**

[1]	F. Kaufmann, G. Finco, A. Maeder, and R. Grange, “Redeposition-free inductively-coupled plasma etching of lithium niobate for integrated photonics,” *Nanophotonics*, vol. 12, no. 8, pp. 1601–1611, 2023.

[2]	C. Jin *et al.*, “Fabrication of lithium niobate metasurfaces via a combination of FIB and ICP-RIE,” *Chin Opt Lett*, vol. 20, no. 11, p. 113602, Nov. 2022.

[3]	Z. Fang *et al.*, “Monolithic integration of a lithium niobate microresonator with a free-standing waveguide using femtosecond laser assisted ion beam writing,” *Sci. Rep.*, vol. 7, no. 1, p. 45610, Mar. 2017.

[4] David E. Zelmon, David L. Small, and Dieter Jundt, "Infrared corrected Sellmeier coefficients for congruently grown lithium niobate and 5 mol. % magnesium oxide–doped lithium niobate," J. Opt. Soc. Am. B 14, 3319-3322 (1997)

[5] Luigi Moretti, Mario Iodice, Francesco G. Della Corte, Ivo Rendina; Temperature dependence of the thermo-optic coefficient of lithium niobate, from 300 to 515 K in the visible and infrared regions. *J. Appl. Phys.* 1 August 2005; 98 (3): 036101.

[6]	P. Kharel, C. Reimer, K. Luke, L. He, and M. Zhang, “Breaking voltage–bandwidth limits in integrated lithium niobate modulators using micro-structured electrodes,” *Optica*, vol. 8, no. 3, pp. 357–363, Mar. 2021.
